# The CXCL13 hub in obesity-related metabolic inflammation: regulation of spatiotemporal heterogeneity and targeted strategies

**DOI:** 10.1530/JOE-25-0295

**Published:** 2026-04-13

**Authors:** Mengke Huang, Xiaolin Chen, Yongmei Jiang, Ting Liu

**Affiliations:** ^1^ Department of Laboratory Medicine, West China Second University Hospital, and Key Laboratory of Obstetric & Gynecologic and Pediatric Diseases and Birth Defects of Ministry of Education, Sichuan University, Chengdu, China; ^2^ State Key Laboratory of Biotherapy and Cancer Center/National Collaborative Innovation Center for Biotherapy, Sichuan University, Chengdu, China

**Keywords:** CXCL13, obesity, metabolic inflammation, SLO, TLS, FALC

## Abstract

C-X-C motif chemokine ligand 13 (CXCL13) is a crucial regulator of lymphoid tissue development and immune function. It orchestrates homeostasis in secondary lymphoid organs, inflammatory responses in tertiary lymphoid structures (TLSs), and immunometabolic balance within fat-associated lymphoid clusters (FALCs) by mediating the homing of CXCR5^+^ cells and activating stromal cells. In the context of obesity-related metabolic inflammation, CXCL13 acts as a central hub of immunometabolic dysregulation, driving pathological processes across multiple organs with significant organ- and stage-specific heterogeneity, which reflects spatiotemporal heterogeneity. It mediates the pathological transformation of FALCs in adipose tissue, inducing insulin resistance; promotes TLS formation within pancreatic islets, accelerating β-cell destruction; dynamically regulates atherosclerotic plaque stability in the blood vessels; and undergoes a functional shift from compensatory suppression to profibrotic and procarcinogenic roles during the progression of liver disease. The effects of CXCL13 are highly dependent on the local microenvironment, exhibiting both pro-inflammatory and repair-suppressive effects in diabetic complications. Although targeted therapies show experimental promise, the context-dependent functions of CXCL13 – encompassing both physiological protection and pathological disruption – along with its organ and stage specificity, necessitate spatiotemporally precise interventions. This review systematically elucidates the pivotal role of CXCL13 in obesity-related metabolic inflammation, providing a theoretical foundation for the development of precision intervention strategies tailored to disease subtypes, stages, and specific organ targeting.

## Introduction

Chemokines are a class of structurally highly conserved small peptide molecules. Based on the position of cysteine residues at their N-terminus, they are classified into four subfamilies: CXC, CC, XC, and CX3C (1, 2). These molecules are secreted by various cell types upon induction or are constitutively expressed (3). C-X-C motif chemokine ligand 13 (CXCL13), also known as B cell-attracting chemokine 1 or B lymphocyte chemoattractant, is a homeostatic chemokine that belongs to the CXC subgroup and represents the most potent chemoattractant for B cells (4, 5). C-X-C chemokine receptor type 5 (CXCR5), also referred to as Burkitt’s lymphoma receptor 1, is the sole known receptor for CXCL13 (5). It is primarily expressed on B cells, follicular helper T (Tfh) cells, and migratory dendritic cells (DCs) (6, 7). Tfh cells are specialized helpers for B cells, supporting B cell differentiation, antibody production, and germinal center (GCs) reactions through cytokines and co-stimulatory signals (8).

Under physiological conditions, CXCL13 is primarily expressed by stromal cells and Tfh cells, serving as a key regulator of lymphoid tissue structure development and immune function (9, 10). It directs the homing of B cells and Tfh cells to the follicular zones of secondary lymphoid organs (SLOs) (including lymph nodes, spleen, tonsils, Peyer’s patches, and mucosa-associated lymphoid tissue) (9, 11). CXCL13 collaborates with C-C motif chemokine ligands 19 and 21 (CCL19 and CCL21) to establish T–B cell compartmentalization, promotes B cell positioning within GCs, facilitates T–B cell interactions, and supports antibody affinity maturation (12, 13, 14). Within body cavities (such as the peritoneal and pleural cavities), macrophages and mesothelial cells constitutively express CXCL13, recruiting B1 cells to home to fat-associated lymphoid clusters (FALCs) (15, 16). This process mediates the production of natural IgM antibodies, providing early immune defense (16, 17). Studies using CXCL13 gene-deficient models (CXCL13^−/−^ mice) have confirmed its essential physiological functions, demonstrating SLO hypoplasia and defective localization and function of B1 cells within FALCs (18, 19).

Under chronic inflammatory stimuli, such as autoimmune diseases, infections, and tumors, activated stromal cells, T peripheral helper (Tph) cells, and macrophages in non-lymphoid tissues exhibit aberrant CXCL13 overexpression – termed ectopic CXCL13 expression (12, 20, 21). This induces elevated plasma CXCL13 levels correlating with inflammatory activity (22, 23). Ectopic CXCL13 collaborates with chemokines such as CCL21 to recruit CXCR5^+^ lymphocytes to non-lymphoid tissues, driving the formation of tertiary lymphoid structures (TLSs) containing GCs (24, 25). TLSs initiate local immune responses independently of SLOs: they confer protection by enhancing pathogen clearance during infections, yet promote pathology by exacerbating tissue damage in chronic inflammation (26). CXCL13 levels positively correlate with TLS maturity and disease severity, while CXCR5 deficiency abolishes TLS formation in mice (27). Notably, FALCs in visceral adipose tissue (VAT) undergo TLS-like remodeling upon inflammatory stimulation, characterized by increased number, size, and GC formation (28).

CXCL13, a key mediator of chronic inflammation, is increasingly recognized as a central hub in obesity-associated metabolic inflammation. Obesity – defined as a body mass index (BMI) ≥ 30 kg/m^2^ – affects over 1 billion people worldwide (29). Its core pathology involves chronic low-grade systemic inflammation triggered by abnormal adipose tissue expansion (30). This unique metabolic inflammation (distinct from infectious or autoimmune inflammation) persistently activates innate immunity, disrupts metabolic homeostasis, and targets organs including the pancreas, liver, blood vessels, and adipose tissue (31, 32, 33). Consequently, it drives the development of type 2 diabetes mellitus (T2DM), nonalcoholic fatty liver disease (NAFLD), and atherosclerosis (AS) (34, 35, 36). Within the obese microenvironment, infiltrating immune cells dysregulate chemokine networks (including CXCL13, CCL19, and CCL21), promoting pathological TLS neogenesis or aberrant FALC expansion, thereby exacerbating tissue damage (31, 37). The profound crosstalk between inflammatory and metabolic signaling offers new perspectives on obesity-related multi-organ complications. A comprehensive elucidation of the dynamic regulation of CXCL13 in lymphoid structure formation and organ-specific inflammation is essential for unraveling the pathological heterogeneity of obesity-associated diseases and advancing precision interventions.

## CXCL13: the orchestrator of constitutive and inducible lymphoid structures

CXCL13 functions as a crucial regulatory chemokine that orchestrates lymphoid immune functions by governing the development and operation of various lymphoid structures through the recruitment of CXCR5^+^ immune cells and the activation of stromal cell networks. These structures are categorized into three main types based on their developmental origins and microenvironmental characteristics: embryonically programmed SLOs, inflammation-induced ectopic TLSs, and FALCs residing in metabolically active VAT (Table 1). CXCL13 exhibits distinct regulatory patterns across these structures: it maintains homeostatic architecture in SLOs, drives inflammatory cascades in TLSs, and precisely regulates immunometabolic switching in FALCs. This functional heterogeneity provides critical mechanistic insights into the immunopathology of obesity-associated metabolic inflammation while establishing a foundation for precision-targeted therapeutic strategies against CXCL13.

**Table 1:** CXCL13-mediated heterogeneity in lymphoid structures: comparative analysis of developmental and functional features across SLOs, TLSs, and fat-associated lymphoid clusters.

Characteristics	SLOs	TLSs	FALCs
Structural organization	Highly structured: contains GCs, distinct T/B cell zones, FDC networks, FRC networks, conduits, HEVs, and a complete lymphatic vessel system	Highly structured: contains GCs, distinct T/B cell zones, FDC networks, FRC networks, and HEVs; may partially develop conduits and lymphatic vessels	Steady state: loose cellular aggregates lacking GCs, distinct T/B zones, FDC networks, HEVs, conduits, and lymphatic vessels
			Inflammation/infection: develops GCs, T/B zones, and HEVs; remains devoid of FDC networks, conduits, and lymphatic vessels
Capsule presence	Present in spleen, lymph nodes, and tonsils (physically separating them from the tissue microenvironment); absent in PPs and MALTs (directly exposed to the tissue microenvironment)	Absent (directly exposed to the local inflammatory microenvironment)	Absent (directly exposed to the tissue microenvironment)

Distribution	Systemically distributed	Chronic inflammatory foci within non-lymphoid organs/tissues (under persistent inflammation)	Within visceral VAT (e.g., greater omentum and mesentery)
Developmental timeline	Embryonically programmed (pre-established developmental pathways)	Postnatally induced by persistent local inflammatory signals (within affected tissues)	Steady state: postnatally induced, shaped by commensal microbiota
			Inflammation/infection: driven by local inflammatory signals
Formation mechanism	Dependent on CXCL13 and LTβR signaling secreted by LTi cells and LTo cells	Dependent on CXCL13 and LTβR signaling secreted by mature immunofibroblasts	Dependent on CXCL13 signaling secreted by stromal cells and macrophages, coordinated by ILC2s

Primary functions	Systemic adaptive immunity or mucosal immune defense; clearance of self-reactive lymphocytes	Localized adaptive immune responses in non-lymphoid tissues (under chronic inflammation); defective peripheral tolerance with autoantibody production	Localized adaptive immune responses within serosal cavities (e.g., peritoneal and pleural spaces)

Clinical implications	Foundation of systemic immune homeostasis	Context-dependent disease modulator: protective roles: anti-tumor and anti-infection immunity	Regulation of immunometabolic homeostasis and serosal cavity immune defense
		Pathological roles: aggravation of autoimmune disorders	

SLO, secondary lymphoid organ; TLS, tertiary lymphoid structure; FALC, fat-associated lymphoid cluster; GCs, germinal centers; FDC, follicular dendritic cell; FRC, fibroblastic reticular cell; HEVs, high endothelial venules; PPs, Peyer’s patches; MALTs, mucosa-associated lymphoid tissues; VAT, visceral adipose tissue; LTi, lymphoid tissue inducer; LTo, lymphoid tissue organizer; and ILC2s, innate lymphoid cells type 2.

### SLOs

**Figure 1:**
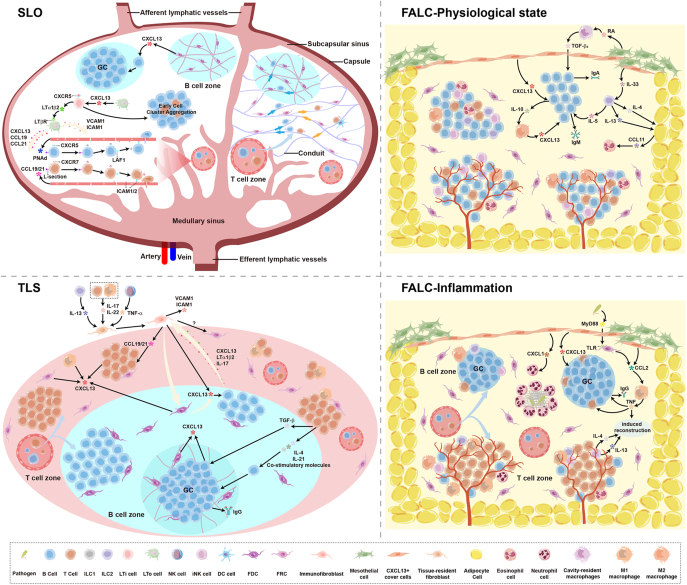
CXCL13 dynamically regulates the functions of three types of lymphoid structures: it maintains homeostatic architecture in SLOs, drives inflammatory ectopic aggregation in TLSs, and mediates the transition of fat-associated lymphoid clusters from physiological barrier protection to inflammatory immune responses. A full color version of this figure is available at https://doi.org/10.1530/JOE-25-0295.

SLOs are permanent structures developmentally programmed during embryogenesis, providing the essential architectural foundation for systemic immune responses. Their development initiates with lymphoid tissue organizer (LTo) cells – also termed stromal organizer cells – which secrete CXCL13 to recruit CXCR5-expressing lymphoid tissue inducer (LTi) cells, forming primitive cell clusters (Fig. 1, SLO) (18). The lymphotoxin alpha1/beta2 heterotrimer (LTα1β2) on LTi cell surfaces binds to the lymphotoxin beta receptor (LTβR) on LTo cells, activating a core positive feedback loop. This signaling upregulates the expression of vascular cell adhesion molecule 1 (VCAM1), intercellular adhesion molecule 1 (ICAM1), and chemokines (including CXCL13, CCL19, and CCL21) by LTo cells (38). This process not only sustains recruitment and retention of immune cells but also induces differentiation of LTo cells into stromal subpopulations such as follicular dendritic cells (FDCs) and fibroblastic reticular cells (FRCs) (38, 39). Ultimately, mature SLO structures emerge, including GCs, segregated T/B cell zones, high endothelial venules (HEVs), and fully developed lymphatic vessel systems.

Continuous lymphocyte circulation between the bloodstream and SLOs is mediated by two specialized vascular systems: HEVs and lymphatic vessels. In mature SLOs (excluding the spleen), lymphocyte infiltration primarily occurs through HEVs via a tightly regulated multistep cascade (40). First, peripheral node addressin (PNAd) expressed on HEVs binds to leukocyte selectin (L-selectin) on lymphocytes, mediating rolling adhesion (41, 42). Subsequently, CXCL13 (predominantly secreted by stromal cells) and CCL21 (mainly produced by endothelial cells) presented on the luminal surfaces of HEVs activate the lymphocyte integrin lymphocyte function-associated antigen 1 (LFA-1), promoting its binding to endothelial intercellular adhesion molecules ICAM-1 and ICAM-2 for firm adhesion (43, 44). Ultimately, lymphocytes transmigrate across the endothelium into the SLOs. Lymphocyte positioning within SLOs is finely regulated by chemokine gradients. CXCL13 guides naïve B cells to follicles along FRC networks via CXCR5, while CCL21 and CCL19 direct naïve T cells to T-cell zones along FRC networks via C-C motif chemokine receptor 7 (CCR7) (45, 46). In this process, stromal cells execute essential organizational functions. FRCs in T-cell zones form conduit systems that coordinate T-cell migration, dendritic cell interactions, and the transport of small antigens (47, 48). FDCs constitutively express high levels of CXCL13, which is crucial for B-cell retention in follicles, B-cell activation, and GC reactions (including somatic hypermutation and class switching) (4, 49, 50). Additionally, the lymphatic vessels throughout the entire SLOs form a complete dynamic drainage network – ranging from afferent lymphatics to subcapsular sinuses to efferent lymphatics – efficiently performing the functions of transporting and recycling antigens and specific immune cells, mainly migratory DCs, along with a small number of naïve T cells and effector memory T cells (46, 51).

Under inflammatory stimulation, SLOs undergo adaptive remodeling characterized by HEV expansion and upregulated adhesion molecule expression, promoting massive infiltration of naïve lymphocytes, effector T cells, and monocytes (52). Simultaneously, increased afferent lymphatic flow delivers abundant mature DCs, while lymphocyte egress obstruction synergistically expands the SLO immunocyte reservoir, significantly enhancing antigen presentation efficiency (53). Notably, the inflammatory microenvironment induces CXCL13 upregulation that accelerates B-cell follicular aggregation, thus augmenting immune surveillance capacity (18). As central hubs of immune homeostasis, SLOs utilize highly specialized stromal cells to precisely orchestrate key molecules, including CXCL13, directing orderly immune cell migration, positioning, and interactions – this dynamically tunable regulatory scaffold provides a central reference framework for understanding TLS formation induced by inflammation.

### TLSs

TLSs are transient ectopic lymphoid aggregates developing within non-lymphoid tissues in response to chronic inflammatory stimuli – such as autoimmune diseases, infections, cancer, or aging – that typically resolve upon inflammation subsidence (54, 55). Although exhibiting SLO-like architectural features, including GCs, segregated T/B cell zones, FDC networks, and HEVs, they lack fibrous capsules and complete lymphatic vessel systems (56). As a result, they are directly exposed to antigens and damage signals in local inflammatory microenvironments, which may provoke heightened regional immune responses (57). CXCL13 serves as the core organizer of TLS formation, orchestrating the entire process from initiation and expansion to functional maturation.

TLS formation initiates with inflammatory reprogramming of tissue-resident fibroblasts. Prolonged stimulation of these fibroblasts by cytokines, including interleukin-13 (IL-13), interleukin-17 (IL-17), interleukin-22 (IL-22), and tumor necrosis factor alpha (TNF-α), leads to their transformation into immunofibroblasts (Fig. 1, TLS) (58, 59). Functionally analogous to LTo cells in SLOs, these immunofibroblasts highly express chemokines (CXCL13, CCL19, and CCL21) and adhesion molecules (VCAM-1 and ICAM-1), recruiting naïve B and T cells to sites of inflammation (20, 21, 37, 60, 61). The recruited B cells secrete CXCL13 and synergize with LTα1β2 and IL-17 to activate the non-canonical nuclear factor kappa B (NF-κB) pathway, driving immunofibroblast proliferation and differentiation into FDCs (56, 62). The formation of FDCs further amplifies CXCL13 secretion, establishing a self-reinforcing positive feedback loop that accelerates TLS maturation. Additionally, immunofibroblasts may differentiate into FRCs supporting T-cell activation, although their differentiation mechanisms remain incompletely elucidated.

Lymphocyte aggregate expansion depends on synergistic interactions among diverse immune cells. In this process, the secretion of CXCL13 by stromal cells – including immunofibroblasts and FRCs – initiates lymphocyte clustering (63). Subsequently, recruited macrophages, T cells, and nascent FDCs become essential supplementary CXCL13 sources (55, 56). The CXCL13–CXCR5 axis selectively recruits B cells, Tfh cells, and macrophages, while these recruited cells feedback-secrete CXCL13, establishing a transcellular cooperative positive feedback circuit that continuously amplifies the chemokine gradient, promoting further lymphocyte aggregation and structural maintenance (12, 64, 65, 66). Specifically, B cells persistently produce CXCL13 within follicles, whereas T cells enhance CXCL13 expression through factors such as transforming growth factor beta (TGF-β) (67). Within these aggregates, Tfh cells directly drive B-cell differentiation into GC B cells through interleukin-4 (IL-4), interleukin-21 (IL-21), and co-stimulatory molecules, facilitating antibody affinity maturation (68, 69). Notably, due to absent central tolerance mechanisms, autoreactive B cells generated therein fail to be effectively eliminated, predisposing to pathogenic autoantibody production.

The structural maturation of TLSs endows them with adaptive immune functionality. Hallmarks of mature TLSs include the formation of functional HEVs and networks of FDCs within GCs. HEVs develop through the transformation of local vascular endothelial cells under the influence of LTβR signaling (70). These vessels mediate the continuous infiltration of lymphocytes from the bloodstream to sustain chronic inflammation (70). At the mature stage, FDCs emerge as the primary producers of CXCL13 within GCs (63). They develop via LTβR and tumor necrosis factor receptor (TNFR) signaling pathways, constitutively expressing high levels of CXCL13 to retain B cells and capture antigens, thereby supporting GC reactions (56, 62). Although ductal and lymphatic structures are present in some mature TLSs, their function relies primarily on HEV-mediated cell infiltration and local chemokine concentration gradients due to the absence of a complete drainage network (71, 72). Ultimately, mature TLSs establish a functional local immune microenvironment capable of antigen presentation (mediated chiefly by DCs and B cells), lymphocyte activation, clonal expansion, and memory formation, thus executing local adaptive immune responses (72, 73).

The presence of TLSs is a hallmark of persistent chronic inflammation, and their functionality is highly dependent on the disease context. In infections or tumors, TLSs contribute to pathogen clearance and anti-tumor immunity by enhancing local antigen presentation efficiency and antibody production (72). Conversely, in autoimmune diseases, this localized adaptive immune response – particularly the production of autoantibodies – exacerbates tissue damage (23). Consequently, CXCL13, a key driver of TLS formation and maintenance, exhibits expression levels that directly correlate with structural maturity, inflammation severity, and disease progression (63). Targeting the CXCL13–CXCR5 axis has thus emerged as a potential therapeutic strategy for intervening in TLS development, modulating local immune responses, and treating associated diseases.

### FALCs

FALCs are specialized lymphoid structures constitutively distributed beneath the serosal lining of VAT – such as the greater omentum and mesentery – under physiological conditions (74, 75). Similar to SLOs, FALCs mediate local immune surveillance and early immune responses in the steady state. However, their architecture differs significantly from SLOs: FALCs lack a fibrous capsule, do not form GCs, and are devoid of distinct T/B cell zones, FDC networks, HEVs, conduits, and lymphatic vessels (76, 77, 78, 79). Yet, they possess a highly vascularized structure that supports rapid immune cell recruitment and infiltration (80). The core region of FALCs is enveloped by a monolayer of specialized CXCL13^+^ mesothelial cells, known as FALC cover cells (Fig. 1, FALC – physiological state) (81). This core is enriched with clusters of B1 cells and supported by a network scaffold formed by CCL19^+^ FRCs (82). It also contains T cells, innate lymphoid cells type 2 (ILC2s), M2 macrophages, eosinophils, and invariant natural killer T (iNKT) cells, collectively establishing a homeostatic immune niche dominated by type 2 immune responses (82, 83). FALC formation relies on a unique mechanism dependent on ILC2s and CXCL13 signaling from stromal cells (particularly FALC cover cells) and macrophages, contrasting with the LTi cells and LTβR signaling pathway essential for SLO development (15, 19, 84, 85, 86). Constitutive CXCL13 secretion by cover cells is crucial for maintaining B1 cell positioning and survival within the core (19). Concurrently, interleukin-33 (IL-33) release from cover cells activates neighboring ILC2s (87). Upon activation, ILC2s drive B1 cell proliferation and IgM secretion via interleukin-5 (IL-5) production and induce C-C motif chemokine ligand 11 (CCL11) expression in adipose stromal cells through IL-4 and IL-13 secretion, thereby recruiting eosinophils (76, 87, 88). This cascade, orchestrated by FALC cover cells, CXCL13, and ILC2s, collectively contributes to metabolic balance and immune homeostasis within VAT.

Upon inflammatory or infectious stimulation, FALCs undergo rapid remodeling (Fig. 1, FALC – inflammation). Their number and volume increase dramatically, and they develop GCs, relatively distinct T/B cell zones, and HEVs, exhibiting features resembling TLSs but still lacking FDC networks (28). During this process, FRCs sense pathogen-associated molecular patterns through the Toll-like receptor adaptor protein myeloid differentiation primary response 88 (MyD88) and secrete C-C motif chemokine ligand 2 (CCL2) to recruit inflammatory monocytes (80). FRCs and the recruited M1 macrophages promote T–B cell interactions via TNF–TNFR signaling, thereby driving GC reactions and antibody production (80). Concurrently, FALC cover cells secrete CCL2 to recruit M1 macrophages and C-X-C motif chemokine ligand 1 (CXCL1) to recruit neutrophils (81). These neutrophils form neutrophil extracellular trap-like aggregates within FALCs, characterized by an outer DNA structure expressing citrullinated histone H3, which efficiently capture luminal pathogens (81). The newly formed HEVs further accelerate the sustained infiltration of peripheral immune cells (77). TNF signals from infiltrating M1 macrophages, combined with IL-4 and IL-13 secreted by iNKT cells, collectively induce the generation of new FALCs (28). These changes drive the shift in the FALC microenvironment from a type 2 immunity-dominated steady state toward a type 1 inflammatory state, characterized by expansion of M1 macrophages, CD8^+^ T cells, T helper 1 (Th1) cells, neutrophils, and activated B cells, concomitant with a relative decline in resident type 2 immune cells (89).

CXCL13, constitutively expressed by FALC cover cells and macrophages throughout both physiological homeostasis and inflammatory responses, serves as a central hub for maintaining FALC structural integrity and lymphocyte recruitment. Under steady-state conditions, CXCL13 derived from FALC cover cells orchestrates type 2 immune cells, such as ILC2s and B1 cells, to collectively maintain metabolic balance and immune homeostasis. Conversely, during inflammation, CXCL13 secretion drives the production of divergent cytokines that promote immune cell infiltration and type 1 immune responses, consequently leading to immune–metabolic imbalance. This bidirectional core function in physiological surveillance and inflammatory remodeling underscores the unique role of CXCL13 within FALCs. Given the critical involvement of FALCs in obesity-associated inflammation, peritoneal infections, and autoimmune diseases, targeting the CXCL13–CXCR5 axis may present a novel therapeutic strategy for disorders at the metabolic–immune interface.

## CXCL13-driven organ-specific inflammatory cascades: the heterogeneous network underlying obesity-associated multi-organ pathology

**Figure 2:**
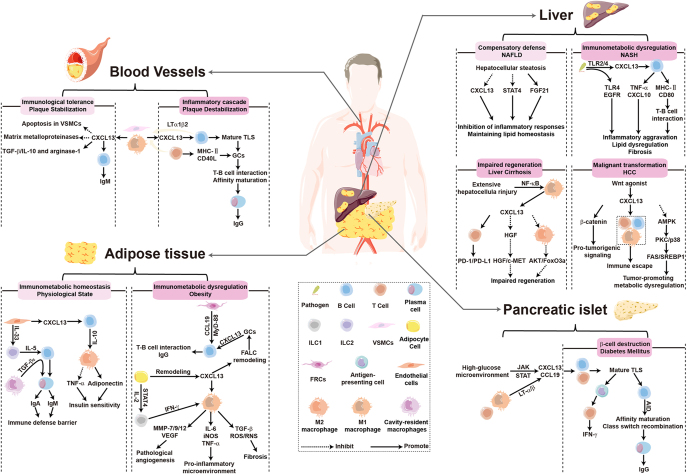
Biphasic spatiotemporal regulation of the CXCL13 hub in obesity-related immunometabolism: orchestrating the activation and migration of immune cells (T/B lymphocytes and macrophages), inducing the formation of TLSs and remodeling of fat-associated lymphoid clusters, and triggering inflammation–fibrosis cascades in different organs. This dynamic network facilitates a functional shift from early metabolic protection to late-stage organ damage. A full color version of this figure is available at https://doi.org/10.1530/JOE-25-0295.

Under obesity-associated metabolic dysregulation, the microenvironment of lymphoid tissues – previously in a homeostatic or controlled state – undergoes profound alterations. This results in a shift in the role of CXCL13 from maintaining immune homeostasis to driving a sustained inflammatory cascade, thereby contributing to the formation of heterogeneous pathological networks in organs affected by chronic inflammation. The persistent accumulation of obesity-induced chronic low-grade inflammation across multiple organs serves as the central driver of metabolic syndrome and its complications, including insulin resistance, T2DM, AS, NAFLD, and its malignant transformation. As a critical nexus linking metabolic dysregulation and immune imbalance, CXCL13 plays a pivotal role in this systemic pathological process. Therefore, it is essential to elucidate its precise regulatory networks within specific organ microenvironments and disease stages (Fig. 2). This undertaking is crucial for deciphering the heterogeneous mechanisms underlying obesity-related multisystem complications and for developing targeted therapies.

### Adipose tissue

Adipose tissue, acting as a central hub in obesity-associated metabolic inflammation, undergoes a distinctive transformation in its FALC structure, revealing a CXCL13-driven immunometabolic cascade that progresses from maintaining physiological homeostasis to forming a pathological conversion characterized by features of TLSs, a process directly leading to systemic metabolic disorders such as insulin resistance (Fig. 2, adipose tissue). Under physiological conditions, FALCs in VAT establish a unique immune microenvironment that maintains immune homeostasis and metabolic balance through a CXCL13-dependent dual immunoregulatory axis. First, CXCL13^+^ mesothelial cells constitutively express high levels of CXCL13, continuously recruiting anti-inflammatory B1 cells to colonize FALCs (19). These B1 cells secrete interleukin-10 (IL-10), which inhibits pro-inflammatory Ly-6C^hi^ monocyte retention while promoting anti-inflammatory Ly-6C^low^ monocyte accumulation (90). Concurrently, in concert with M2 macrophages, they reverse TNF-α-mediated inhibition of insulin signaling pathways and stimulate adiponectin secretion, thereby maintaining local insulin sensitivity (91, 92). Second, IL-33 derived from CXCL13^+^ mesothelial cells activates ILC2s, which in turn secrete IL-5 to promote the proliferation and differentiation of B1 cells into IgM plasma cells (88). Furthermore, retinoic acid stimulates cavity-resident macrophages to produce TGF-β_2_, promoting IgA class switching in B1 cells to establish an immune barrier against commensal bacteria (82). At this stage, the FALC microenvironment – dominated by type 2 immunity components including ILC2s, M2 macrophages, and B1 cells – forms the immunological cornerstone of metabolic equilibrium (93).

Obesity-induced adipose tissue remodeling, characterized by extracellular matrix stiffening and adipocyte hypertrophy, directly stimulates CXCL13 secretion from adipocytes (94, 95, 96). Although highly expressed in mature adipose tissue, CXCL13 does not directly participate in adipogenesis but instead drives pathological progression through immune regulation (97, 98). This adipocyte-derived CXCL13 activates M1 macrophages via paracrine signaling and, in concert with iNKT cells, induces abnormal expansion in both the number and size of FALCs through TNF, IL-4, and IL-13 signaling pathways (19, 82, 99, 100). During early high-fat diet-induced obesity, subcutaneous adipose tissue promotes innate lymphoid cells type 1 (ILC1s) proliferation and interferon gamma (IFN-γ) secretion via interleukin-12 (IL-12) and signal transducer and activator of transcription 4 (STAT4) signaling, directly activating M1 macrophages (101). These activated M1 macrophages enhance angiogenesis through secretion of matrix metalloproteinases (MMPs) (including MMP-7, MMP-9, and MMP-12) and vascular endothelial growth factor (VEGF), while simultaneously exacerbating fibrosis via release of TGF-β, reactive oxygen species, and reactive nitrogen species (94, 102, 103). By disrupting the protective IL-10 network, they establish a pro-inflammatory microenvironment centered on TNF-α, interleukin-6 (IL-6), and inducible nitric oxide synthase (iNOS), ultimately inducing systemic insulin resistance (74). Concurrently, CXCL13 drives pro-inflammatory phenotypic transformation in endothelial cells, upregulating adhesion molecules to increase vascular permeability and facilitate immune cell infiltration (99, 100). Sustained inflammatory stimulation drives the transformation of FALCs into structures resembling TLSs. Within FALCs, CXCL13 recruits B2 cells into GCs (104). Acting in concert with the CCL19–MYD88 signaling pathway in FRCs, this recruitment facilitates CD4^+^ T cell co-localization, thereby promoting T–B cell interactions and IgG class switching (79). This cascade culminates in increased secretion of autoreactive IgG antibodies, exacerbating inflammation in VAT and contributing to insulin resistance. Activated fibroblasts further support B cell activation by constructing a three-dimensional stromal network through collagen and fibrin secretion, simultaneously amplifying inflammatory signals and accelerating the pathological process (103). Concurrently, chronic inflammation persistently depletes B1 cells, further impairing their critical anti-inflammatory and barrier functions (92).

Ultimately, a self-perpetuating cycle is established: adipose tissue remodeling stimulates CXCL13 secretion, which perpetuates chronic inflammation and drives the rapid remodeling of FALCs, thereby accelerating the pathological progression of insulin resistance and systemic metabolic disorders. This process reveals a CXCL13 gradient imbalance across tissues: serum CXCL13 levels are significantly reduced in obese patients, positively correlating with high-density lipoprotein and negatively correlating with cholesterol and low-density lipoprotein (LDL) (105). This reduction reflects impaired systemic immune chemotactic function. Conversely, locally elevated CXCL13 levels in mouse peritoneal ascites highlight the predominance of tissue-specific macrophage recruitment (106). Collectively, this disrupted CXCL13 gradient constitutes a core mechanism of immunometabolic homeostasis dysregulation, whereby systemic concentration imbalance exacerbates lipid metabolism disorders, while local pathological elevation drives a vicious cycle of inflammation – conclusively demonstrating the mechanism underlying immunometabolic equilibrium disruption.

### Pancreatic islets

As a key target organ in obesity-associated chronic inflammation, pancreatic islets experience β-cell dysfunction that acts as the core driver of diabetes development and progression. Obesity-induced metabolic dysregulation and systemic inflammation create the foundation for aberrant CXCL13 expression, establishing it as a critical molecular nexus linking obesity-related inflammation, pancreatic islet damage, and diabetic complications. Within the pancreatic islet microenvironment, CXCL13 functions as a central factor driving inflammatory cell accumulation and β-cell destruction (Fig. 2, pancreatic islet) (107). Furthermore, CXCL13 serves as a key inducer of TLS formation within pancreatic islets, particularly in autoimmune-mediated pancreatic islet damage (107). Clinical evidence demonstrates a positive correlation between CXCL13 levels, added sugar intake, and the incidence of T2DM (108). The expression of CXCL13 is regulated by high-glucose-activated pathways, such as Janus kinase and STAT, thereby amplifying systemic low-grade inflammation (109, 110). Obesity- or autoimmunity-mediated inflammatory infiltration (predominantly involving CD8^+^ T cells and macrophages) induces local upregulation of LT-α/β, subsequently triggering CXCL13 and CCL19 expression (111, 112). This chemokine-mediated recruitment of B and T cells drives the progressive formation of mature TLSs featuring organized structures, including GCs, FDCs, and HEVs (113, 114, 115, 116). Within mature TLSs, immune cells – primarily B cells, plasma cells, and antigen-presenting cells – cooperatively destroy β-cells through multiple mechanisms. CXCR5^+^ B cells in GCs utilize activation-induced cytidine deaminase (AID) to mediate antibody affinity maturation and class switch recombination, thereby driving autoreactive B cell proliferation (117). CD138^+^ plasma cells directly attack β-cells via local secretion of anti-insulin autoantibodies (118). CD45^−^ MHC-II^+^ non-hematopoietic cells activate infiltrating CD4^+^ T cells through pancreatic islet antigen presentation, inducing secretion of pro-inflammatory factors, such as IFN-γ, that promote β-cell apoptosis (115). Crucially, TLS maintenance relies on persistent antigenic stimulation from residual β-cells. Extensive β-cell depletion reduces inflammatory signals (including CXCL13), consequently diminishing the incidence of functionally mature TLSs and immune cell infiltration (114, 118, 119). This dynamic clearly demonstrates the direct association between CXCL13-driven immune restructuring and pancreatic islet destruction progression. Such cumulative pancreatic islet damage and inflammatory processes ultimately contribute to diabetes development and progression.

Furthermore, with diabetes progression, CXCL13 – functioning as a pivotal mediator at the intersection of obesity-related metabolic disorders (hyperglycemia and hyperlipidemia) and chronic inflammation – demonstrates strongly compartmentalized expression and functionality across diabetic complications. The heterogeneous tissue-specific expression patterns of CXCL13 underscore its critical dependence on local microenvironments. Upregulation occurs predominantly in inflammation-driven complications, including diabetic retinopathy (DR), diabetic foot ulcer (DFU), painful diabetic neuropathy (PDN), and tendinopathy. In DR, Tfh cells secrete CXCL13 to promote B-cell proliferation and activation, exacerbating vascular leakage and neovascularization; CXCL13 levels show significant positive correlations with fasting glucose, glycosylated hemoglobin type A1c (HbA1c), and BMI (120). In DFU, CXCL13 overexpression activates IL-17 and TNF signaling pathways, driving excessive immune cell infiltration that potentiates inflammation (121). In PDN, neuronal CXCL13 activates astrocytes through extracellular signal-regulated kinase (ERK), STAT3, and protein kinase B (AKT) signaling cascades, inducing release of TNF-α, IL-6, and interleukin-1β (IL-1β), which collectively mediate central sensitization and neuropathic pain (122). In tendinopathy, glucolipotoxicity induces senescence in tendon-derived stem cells (TSCs) while aberrantly activating CXCL13, thereby promoting TSCs’ osteogenic differentiation that ultimately impairs tendon repair and causes ectopic ossification (123). In complications characterized by impaired repair mechanisms – diabetic peripheral neuropathy (DPN) and diabetic bone defects – CXCL13 expression is downregulated. In DPN, diminished CXCL13 disrupts immune cell infiltration balance (notably neutrophils, macrophages, B cells, and NK cells), exacerbating neural damage through dysregulated inflammation (124). Similarly, hyperglycemia in diabetic bone defects suppresses CXCL13, concurrently inhibiting bone marrow-derived stromal cells (BMSCs) proliferation, promoting apoptosis, and reducing alkaline phosphatase activity (125, 126). These alterations collectively impair osteogenic capacity – a pathological process reversible upon CXCL13 restoration. This context-dependent duality originates from tissue-specific microenvironmental interpretations of CXCL13 signaling under metabolic stress, ultimately polarizing outcomes toward either pro-inflammation or repair suppression. Critically, CXCL13 serves dual pathogenic roles: as a primary driver of pancreatic islet inflammation and TLS formation that directly facilitates β-cell destruction and as a metabolic–inflammatory nexus propagating multi-organ crosstalk. Its organotypic expression patterns thus bidirectionally modulate the trajectories of diabetic complications.

### Blood vessels

The blood vessels, as a principal target in obesity-associated chronic inflammation, undergoes AS progression characterized by chronic inflammatory responses to dysregulated lipoprotein metabolism. This process entails aberrant activation of vascular smooth muscle cells (VSMCs), fibroblasts, and immune cells (such as B cells, T cells, and M1 macrophages) (127, 128). Within the obesogenic environment characterized by hyperlipidemia and chronic low-grade inflammation, CXCL13 mediates stage-dependent regulatory functions throughout AS pathogenesis (Fig. 2, blood vessels). During early plaque stabilization phases, VSMCs and M1 macrophages co-express CXCL13, forming focal enrichments that confer cytoprotective effects. These include suppression of VSMC apoptosis; promotion of anti-inflammatory factor secretion (TGF-β and IL-10) and oxidative stress-mitigating arginase-1; and concurrent inhibition of MMPs expression to preserve fibrous cap integrity (129, 130, 131, 132). Furthermore, locally enriched FoxP3^+^ regulatory T cells (Tregs) attenuate inflammatory cascades while inducing naïve CD4^+^ T-cell differentiation into Tregs (60). These mechanisms synergize with B1 cell-derived oxidation-specific IgM antibodies targeting oxidized LDL, collectively establishing an immune-tolerant vascular niche (129, 132).

Under persistent obesity-associated metabolic inflammatory stimulation and disease progression, CXCL13 undergoes functional transformation that drives plaque destabilization. The CXCL13–CXCR5 axis mediates B-cell migration to the adventitia, facilitating TLS formation. Recruited T/B lymphocytes secrete LTα1β2, which amplifies CXCL13 release from VSMCs and M1 macrophages via positive feedback, promoting FDC network maturation to support GC formation (60, 133, 134). Concurrently, CCL21-recruited T cells drive B-cell plasmacytic differentiation through MHC-II antigen presentation and Tfh-derived CD40L signaling, facilitating affinity-matured production of pro-inflammatory immunoglobulins (e.g., IgG targeting oxidized LDL or heat shock protein 60), thereby exacerbating vascular inflammation (60, 135). Concomitant HEV formation and aberrant lymphoid conduit neogenesis enable direct T/B lymphocytes infiltration, intensifying chemokine/antigen/cytokine crosstalk between vascular layers (135). This shifts TLS immunity toward pro-inflammatory skewing, substantially elevating plaque instability risks. Clinical and experimental evidence supports the stage-specific importance of CXCL13 in the pathogenesis of obesity-related AS. High-fat diets elevate circulating CXCL13 levels in ApoE^−/−^ mice, promoting inflammatory infiltration (136). Mirroring this, human aortic plaques exhibit transient CXCL13 surges specifically during vulnerability phases (137). Clinically, symptomatic carotid atherosclerosis patients show significantly elevated serum CXCL13 following stroke or transient ischemic attack (130). Furthermore, in aged ApoE^−/−^ mice, adventitial TLS size positively correlates with intimal lesion severity (138). Collectively, these findings demonstrate that CXCL13 orchestrates a pathological transition from early immunotolerance to late-stage inflammatory dysregulation, characterized by TLS formation in AS.

### Liver

The liver, a critical target in obesity-associated chronic inflammation, undergoes NAFLD progression driven by persistent metabolic stress and inflammatory signaling. This disease spectrum evolves from simple steatosis through nonalcoholic steatohepatitis (NASH) and fibrosis to hepatocellular carcinoma (HCC). CXCL13 critically drives pathogenesis by mediating immune cell infiltration, disrupting lipid metabolism, and amplifying inflammation (Fig. 2, liver). Its regulatory functions dynamically transition during NAFLD advancement. In the simple steatosis phase, suppressed CXCL13 expression cooperates with attenuated STAT4 signaling and fibroblast growth factor 21 (FGF21) induction to establish a protective network. This coordinated mechanism inhibits inflammatory cascades while preserving lipid homeostasis, constituting the body’s compensatory defense against lipotoxicity (139).

However, when the disease progresses to NASH, intestinal barrier dysfunction results in the leakage of bacterial components via the portal vein, which abnormally upregulates intrahepatic CXCL13 through Toll-like receptor 2/4 (TLR2/4) signaling, thereby chemotactically recruiting B cells to infiltrate the liver parenchyma (140, 141). These infiltrating B cells express high levels of pro-inflammatory factors, such as TNF-α and C-X-C motif chemokine ligand 10 (CXCL10), and enhance their antigen-presenting capacity by upregulating CD80 and MHC-II, thus activating T-cell immune responses (139, 141). This process, in synergy with TLR4 and epidermal growth factor receptor (EGFR) signaling, disrupts the original metabolic compensation, exacerbating chronic inflammation, lipid metabolic disorders, and fibrosis (139, 141). In the cirrhosis stage, persistent liver injury activates macrophages through the non-canonical NF-κB pathway, inducing CXCL13 expression. This suppresses AKT/FoxO3a signaling in Ly-6C^low^ restorative macrophages and downregulates hepatocyte growth factor (HGF) expression, thereby impairing HGF/c-MET pathway-mediated liver regeneration (142). Concurrently, CXCL13^+^ exhausted CD8^+^ T cells form an immunometabolic imbalance network through the activation of programmed cell death protein 1 (PD-1) and programmed death-ligand 1 (PD-L1) immune checkpoint, glycolytic and pyruvate metabolic reprogramming, and dysregulated T-cell receptor (TCR) signaling, thereby cooperatively accelerating cirrhosis progression (143). This dual role of CXCL13 as a ‘switch’ – inhibitory in early stages and activating in late stages – signifies the failure of lipotoxicity defense, constituting the molecular switch that drives the progression from NAFLD to NASH (144).

When the disease progresses to the HCC stage, CXCL13 synergistically drives malignant transformation through three coordinated mechanisms: activating oncogenic pathways, remodeling the tumor microenvironment, and inducing metabolic imbalance. At the molecular pathway level, CXCL13 forms a positive feedback loop with the Wnt/β-catenin pathway – wherein Wnt activators upregulate CXCL13/CXCR5, while CXCL13 inhibitors suppress β-catenin signaling – thereby continuously amplifying oncogenic signals (145). Within the immune microenvironment, CXCL13 recruits CXCR5^+^ CD8^+^ T cells, T helper 17 (Th17) cells, macrophages, and B cells to facilitate immune escape (146, 147, 148, 149). This recruitment promotes IL-12/IL-17 secretion by Th17 cells, exacerbating inflammation-associated carcinogenesis, and induces the production of immunosuppressive IgG4 in B cells (145, 146). Metabolically, CXCL13 suppresses adenosine monophosphate-activated protein kinase (AMPK) phosphorylation while activating the protein kinase C (PKC) and p38 mitogen-activated protein kinase pathway, upregulating lipogenic genes (including fatty acid synthase and sterol regulatory element-binding protein 1) that cause abnormal lipid droplet accumulation, thereby establishing a cancer-promoting metabolic imbalance (150). Clinical evidence further confirms the prognostic significance of CXCL13: levels are markedly elevated in both serum and tumor tissues of advanced HCC patients (145, 151). Serum concentrations positively correlate with liver injury markers, including alanine aminotransferase (ALT) and aspartate aminotransferase (AST), highlighting its potential as a circulating biomarker for HCC progression (145, 151).

## Targeting CXCL13: challenges and prospects for organ- and stage-specific precision interventions

CXCL13 plays a central role in obesity-related metabolic inflammation and cumulative multi-organ damage, making it an exceptionally promising therapeutic target. Current therapeutic strategies targeting CXCL13 primarily involve direct blockade and can be categorized at two levels. At the genetic level, knockout or inhibition of the CXCL13 gene in mice or rats attenuates the inflammatory response (23). In carbon tetrachloride-induced liver injury models, CXCL13 knockout mice exhibited significantly enhanced liver regeneration, as evidenced by reduced serum ALT and AST levels, diminished necrotic areas, and upregulated hepatocyte proliferation markers (such as proliferating cell nuclear antigen and cyclin D1) (142). In tendon abnormality models, targeted inhibition in diabetic mice using small interfering RNA delivered via adeno-associated virus 2 effectively alleviated ectopic ossification process under metabolic disorder conditions (123). In specific *in vitro* cell knockout or inhibition models, targeted knockout of CXCL13 effectively reversed the abnormal osteogenic differentiation of TSCs induced by high glucose and high fat and significantly inhibited the osteogenic differentiation capacity of BMSCs (123, 125). At the protein level, in the liver injury model, blocking CXCL13 with an anti-CXCL13 antibody relieved the suppression of HGF expression by macrophages, activated the HGF/c-MET pathway, promoted hepatocyte proliferation, and thereby accelerated liver regeneration (142). Crucially, however, this ‘beneficial’ effect is not universal: in murine pancreatic islet autoimmune models, anti-CXCL13 antibodies disrupted TLSs within islets but failed to delay diabetes progression (117). This may be because targeting only the CXCL13-driven immune structures may not be sufficient to reverse the CXCL13-mediated functional damage or to adapt to the complex pathological microenvironment, thereby reducing the therapeutic efficacy.

The research above demonstrates that although CXCL13 holds significant potential as a therapeutic target for obesity-related metabolic inflammation, its clinical application still faces fundamental limitations. The function of this chemokine is highly context-dependent and can play completely opposite roles in different target organs and stages of disease development. Moreover, current intervention strategies are mainly limited to specific organs or disease models, posing substantial challenges for clinical application. Within the obesity-associated inflammatory cascade, CXCL13 can exert protective regulation, as seen in early-stage fatty liver or plaque stabilization, while simultaneously acting as a critical switch that drives destructive processes, such as the transition to NASH, plaque destabilization, and the progression of HCC. This double-edged nature demands intervention strategies with high precision (organ-targeted), strict timeliness (disease-stage specificity), and microenvironment adaptability.

Based on the discussion above, future research must urgently focus on two key areas: developing drug delivery technologies capable of crossing biological barriers (such as vascular walls and specific tissues) for precise organ and cell targeting, and exploring combinatorial interventions (such as co-targeting CXCL13 and its synergistic signaling pathways) to counteract complex regulatory networks. Consequently, while the role of CXCL13 as a molecular nexus is undeniable, translating this knowledge into safe and effective clinical therapeutics requires a more in-depth investigation of its finely tuned regulatory mechanisms across the full spectrum of obesity-related inflammatory accumulation and within specific target organ microenvironments.

## Conclusion

This review elucidates the central orchestrating role of CXCL13 in obesity-related metabolic inflammation. Through spatiotemporal heterogeneity, CXCL13 drives pathological progression across multiple organs. This progression manifests spatially as organ-specific lymphoid remodeling, which includes the pathological transformation of adipose tissue FALCs, TLS-induced β-cell destruction in pancreatic islets, dynamic regulation of vascular plaque stability, and malignant transformation from NAFLD to HCC in the liver. Temporally, CXCL13 exhibits stage-dependent functional switching, evolving from early protective maintenance of metabolic homeostasis to late-stage promotion of organ damage. Precisely regulated by local metabolic–immune microenvironments, this dual nature underscores the core challenge of overcoming spatiotemporal heterogeneity for targeted interventions. It necessitates the precise modulation of specific functions in the appropriate organs at optimal time points. Consequently, this review establishes a theoretical framework for developing stratified, staged, and organ-targeted therapies, thereby advancing precision medicine for the multisystem complications of obesity.

## Declaration of interest

The authors declare that this work was conducted in the absence of any commercial or financial relationships that could be construed as a potential conflict of interest.

## Funding

This work was supported by Sichuan Science and Technology Program (2023YFS0186 and 2023YFS0222).

## Author contribution statement

MH wrote the manuscript. XC, YJ, and TL gave valuable suggestions and made critical revisions. YJ and TL conceptualized and supervised the preparation of this manuscript. All authors contributed to the article and approved the submitted version.
